# Aggressive Behaviors among 15–16-Year-Old Romanian High School Students: Results from Two Consecutive Surveys Related to Alcohol and Other Drug Use at the European Level

**DOI:** 10.3390/ijerph17103670

**Published:** 2020-05-22

**Authors:** Adina Bucur, Sorin Ursoniu, Constantin Caraion-Buzdea, Virgil Ciobanu, Silvia Florescu, Cristian Vladescu

**Affiliations:** 1 Department of Public Health, Victor Babes University of Medicine and Pharmacy, 300172 Timisoara, Romania; bucur.adina@umft.ro (A.B.); sursoniu@umft.ro (S.U.); ccaraion@umft.ro (C.C.-B.); ciobanu.virgil@umft.ro (V.C.); 2 Management and Professional Development, The National School of Public Health, 021253 Bucharest, Romania; sflorescu@snspms.ro

**Keywords:** aggressive behavior, adolescents, victimization, bullying

## Abstract

The aim of this paper is to examine aggressive behaviors among Romanian high school students between 15 and 16 years old, to compare data in two national representative samples and to identify factors associated with physical fighting. This study investigates the association of selected factors (social, school performance and substance use) with physical fighting. A total of 2289 Romanian students were included in the 2007 database and 2770 in the 2011 database. This study revealed that 35.87% of the teenagers have taken part in a physical fight during the previous 12 months, as compared with the European average of 31.5%. Romania has the highest prevalence of violent behavior by participating in a group bruising of an individual in both surveys, 2007 and 2011. A logistic regression analysis performed for the 2011 study revealed the following factors associated with physical fighting: binge drinking during the previous 30 days, male gender, serious problems with friends, parent(s) who do not know where and with whom the adolescents spend their evenings, poor parental caring, low school grades, and high truancy. A decrease in almost all aggressive behaviors was noticed in 2011, compared to 2007. These findings may be useful to support and guide policy makers regarding improvement and implementation of strategies to further prevent aggressive behaviors in teenagers.

## 1. Introduction

Aggression in high school students is a problem in many countries [1,2,3,4,5,6,7,8] and adolescents are especially vulnerable to its consequences [9]. Bullying, victimization and fighting illustrate different types of involvement in violence during adolescence. Bullying involves negative physical or verbal action that has hostile intent, causes distress to the victim and includes a power differential between bullies and their victims [6]. According to Olweus, it is also bullying when a person is teased repeatedly in a way he/she does not like. Victimization by bullying occurs when a person is made the recipient of aggressive behavior [10]. Typically, it is someone less powerful than the perpetrator. Fighting is an aggressive behavior and, in most cases, the people involved are of a similar age and equal strength. Demographic, social, academic achievement and substance use (alcohol drinking, tobacco smoking, drug use) have shown association with violent behavior in adolescents [11,12].

According to a report released in 2016, the prevalence of fighting among adolescents aged 15 from Europe and North America varies between 22% and 69% in boys and between 9% and 25% in girls [13].

Physical fighting was strongly associated with alcohol consumption and drug use [14,15,16].

The social developmental model states that the youth behavior is learned through a continuous process starting from childhood. The social agents that play an important role in their behavioral development are families, schools, peers, communities [17,18,19].

Adolescents who maintain a stronger, healthier relationship with their families and their education are less likely to participate in unacceptable behaviors, such as violence [20,21].

The relationship with parents, including poor parental monitoring and low parental support, has also been mentioned as risk factors for violent behavior among adolescents [22,23].

Physical fighting has also been associated with poor peer relationships [24,25]. One of the most important factors in sculpting and defining the adolescent behavior represents the time they spend with their peers and the relationships they establish with them. Numerous studies have shown that adolescents tend to engage in similar behaviors as their peers (smoking, drinking, fighting and/or engaging in sexual behavior) [26,27,28]. Many adolescents have at least one friend that uses substances, but when most of their peers engage in behaviors such as drinking, smoking, or even illegal activities, the risk of them doing the same increases. While engagement in peer group activity is normative for adolescents, it is when a person has high support from peers and low support from parents, that substance use is particularly elevated [28].

Beside the immediate effects, bullying, victimization and fighting have long-term negative consequences for the bullies, victims, fighters and those who observe the interaction [29,30]. Some studies have shown that children who are bullies tend to still be bullies as adults. Additionally, an interesting observation is that adult bullies with children of their own tend to raise them as bullies [30]. Because it relates to students, school violence has received substantial media, research, and political attention [31]. In Romania, no systematic studies on aggressive behavior among high school students have been published so far.

According to the Health Behaviour in School-aged Children (HBSC) study conducted in Romania between 2005 and 2006 [32], 6% of the girls and 24% of the boys aged 15 have been involved in a physical fight at least three times in the last 12 months. Another survey performed by HBSC between 2009 and 2010 [33] found that 4% of the girls and 19% of the boys aged 15 have been involved in a physical fight at least three times in the last 12 months.

However, there is increased concern about violent behaviors amongst adolescents in the school setting and on the community level; hospital, primary health care and ambulatory data shows increased numbers for adolescent victims of aggressive behaviors [34,35]. The 2007 [36] and 2011 [37] European School Survey Project on Alcohol and Other Drugs (ESPAD), which examined 35 and 36 European countries, respectively, including Romania, provided an opportunity to study aggressive behavior in a large national sample.

The first aim of this paper is to examine patterns of aggressive behavior of 15–16-year old high school students in Romania, and compare the collected data in two national representative samples from 2007 and 2011. The second aim of the study is to identify factors (gender, social, behavioral and school performance) associated with physical fights in adolescents in Romania.

We hypothesized that physical fighting is associated with different types of factors, such as demographic (gender), social (relationship with parents and friends, parental control), school performance (grades), problem behavior (truancy) and substance use.

## 2. Materials and Methods

### 2.1. Population, Sampling Design and Representativeness

The ESPAD target population is defined as regular students who turn 16 during the calendar year of the survey and are present in the classroom on the day of the survey [36,37]. This definition includes students who are enrolled in regular, vocational, general or academic studies but excludes those enrolled in either special schools or special classes for students with learning disorders or severe physical handicaps. Part-time and evening students and military high schools were also excluded. Sampling in the ESPAD project is based on the class as the final sampling unit. A total of 104,828 students participated in the 2007 ESPAD study and 103,076 students in the 2011 study. More details about methodology are available in the ESPAD Reports [36,37].

Among all Romanian inhabitants born in 1991 and 1995, roughly 87% and 94%, respectively, were still enrolled in regular schools. The remaining students were enrolled in either a vocational, theological or military school, or in schools where the teaching language is not Romanian.

The Romanian sampling frame included 9th and 10th graders and covered approximately 99% of the ESPAD target population (the remaining students were in the 8th grade). The sampling frame was nationally representative for students from regular schools and covered all 42 counties.

A simple random sampling procedure was applied to a list of 1459 schools in 2007 and 1499 schools in 2011, in order to obtain an adequate geographical distribution. Both lists were provided by the Ministry of Education. These lists did not include information about school size, meaning that all schools had the same probability of being sampled. From these schools, one class per grade was randomly selected to participate without class size being considered. The samples are representative for Romanian students born in 1991 and 1995 enrolled in grades 9 and 10 at regular schools. Using the detailed information about school and class size provided by the schools contacted, a weight has been introduced to adjust for school size.

### 2.2. Organization of the Study

Once classes had been selected, the parents received information about the study in order to give their active consent; the schools received a folder with methodological information and the headmasters were asked to make plans for the data-collection procedure. The questionnaires and response envelopes were distributed by ordinary post to the research assistants.

Research assistants collected data in the classrooms where the students answered the questionnaires anonymously. They received standard instructions and individual sealable response envelopes to put their questionnaires in. The completed questionnaires were brought by the research assistants to the county center where the data were entered.

This study was approved by the Research Ethics Committee of Victor Babes University of Medicine and Pharmacy (No. 03/2013).

### 2.3. School and Student Participation

Students and schools were informed that participation in the survey was voluntary. The overall response rate was 84% in 2007 and 79% in 2011. Only 2% of the research assistants experienced that some of the students found the questionnaire difficult to complete. A total of 2289 Romanian students were included in the final database in 2007 and 2770 in the 2011 database.

### 2.4. Instrument, Measurement and Data Processing

The translation of the questionnaire was made by a team of professional translators, after which it was back-translated and reviewed by a psychiatrist and public-health specialists. The questionnaire was pre-tested at ten schools, which led to some modifications.

Cronbach’s alpha reliability coefficient was determined for the main parts of the questionnaire investigating social support, substance use, violence, etc. Results ranging from 0.77 to 0.81 were found, indicating that participants responded consistently to questionnaire items.

Aggressive behaviors were assessed through the following questions: How many times during the last 12 months he/she had: experienced a physical fight, hit one of the teachers, got mixed into a fight at school or at work, took part in a fight where a group of friends were against another group, hurt somebody badly enough to need bandages or a doctor, used any kind of weapon to get something from a person, participated in a group teasing an individual, participated in a group bruising an individual, participated in a group starting a fight with another group, started a fight with another individual.

Victimization was assessed through questions like: How many times during the past 12 months have you been: individually teased by a whole group of people, bruised by a whole group of people, in a group that was attacked by another group, or individually involved in a fight started by someone else.

The use of tobacco, alcohol and illicit drugs was assessed through questions that aimed to establish whether these substances have ever been used by the participants, age of first use and the possibility of consumption during the past 30 days. Binge drinking was assessed by asking how many days out of the last 30 the respondent had five or more drinks in a row.

The answers for all the question from above were dichotomized to not at all and once or more times.

Relationship with parents and perceived parental behavior were assessed as follows: relationships with parents (satisfied, neither nor, not satisfied, not at all satisfied, there is no such person); family control was assessed by the questions “Do your parent(s) set definite rules about what you can do at home?”, “Do your parent(s) set definite rules about what you can do outside the home?”, “Do your parent(s) know whom you are spending your evenings with?”, “ Do your parent(s) know where you are in the evenings?” (almost always, often, sometimes, seldom, almost never), “Do your parent(s) know where you spend Saturday nights?” (always, quite often, sometimes, usually do not know); emotional support and caring from mother and/or father (almost always, often, sometimes, seldom, almost never).

One item analyzed relationship with friends (satisfied, neither nor, not satisfied, not so satisfied, not at all satisfied, there is no such person) and two items assessed emotional support and caring from best friend (almost always, often, sometimes, seldom, almost never).

Students were also asked about their school performance, mainly their grades at the end of the last term and about absenteeism during last 30 days.

The variable, “How often during the last 12 months have you experienced physical fight?” was dichotomized and the new variable was grouped as follows: never/one or more physical fights during the past 12 months. This question was introduced for the first time in the 2011 survey.

The data were entered manually in each county during a five-week period and then centrally merged by the National School of Public Health, Management and Professional Development, Bucharest, Romania.

### 2.5. Statistical Analysis

All analyses were performed on weighted data. The results are presented as absolute and relative frequencies. All analyses were conducted with Stata 9.2 (Statacorp, Texas, TX, USA) using the svy commands. Descriptive statistics were conducted using frequencies and proportions. Chi-square tests were performed to compare values between 2007 and 2011. A logistic regression analysis was used to estimate factors associated with physical fight experienced during previous 12 months. A *p* < 0.05 was considered statistically significant, and odds ratios (OR) with their respective 95% confidence interval (CI) were calculated.

## 3. Results

A total of 2289 students (1009 males—44.08% and 1280 females—55.92%) were included in the survey in 2007, and 2770 students (1279 males—46.17% and 1491 females—53.83%) in 2011. The present study revealed that 1000 students (35.87%) had experienced a physical fight during the previous 12 months. Univariate analysis showed important differences between students who experienced physical fight and those who did not (Table 1).

The following variables were significant factors associated with physical fighting: male gender, poor relationships with mother and father, parent(s) do not know where adolescents spend their Saturday nights, parent(s) do not know where and with whom the adolescents spend their evenings, poor caring and emotional support from parent(s), serious problems with parents, poor caring and emotional support from best friend, serious problems with friends, low school grades, high truancy, marijuana lifetime use, current smoking and binge drinking during the previous 30 days.

We did not find any association between physical fight experienced during the last 12 months and definite rules set by parent(s) regarding what adolescents can do at home and outside home. Using stepwise logistic regression, the most parsimonious multivariate logistic model was produced for factors associated with physical fight experienced during the last 12 months (Table 2).

The following factors associated with physical fight remained: male gender, parent(s) do not know where and with whom the adolescents spend their evenings, poor parental caring, serious problems with friends, low school grades, high truancy and binge drinking during the previous 30 days. A decrease in almost all aggressive behaviors was noticed in 2011 compared to 2007 (Table 3).

Statistically significant differences were observed for: taking part in a fight where a group of friends went against another group, participating in a group teasing an individual, participating in a group starting a fight with another group (only for females), starting a fight with another individual.

The only violent behavior that increased in 2011, compared to 2007, was using any kind of weapon to get something from a person, but this was statistically significant only for females.

Regarding victimization, we also found a decrease of prevalence in 2011 compared to 2007. Statistically significant differences were observed for: being individually teased by a whole group of people, being bruised by a whole group of people (only for males), being in a group that was attacked by another group, being individually involved in a fight started by someone else.

## 4. Discussion

The ESPAD surveys among high school students provided us an opportunity to study aggressive behavior in two large national samples in 2007 and 2011. The present study revealed that 35.87% of the students experienced physical fight during the previous 12 months. This result was higher than the prevalence of physical fight for the entire European ESPAD database [38] that was 31.25%, the lowest value being recorded in Denmark (19.68%) and the highest in Malta (47.74%) [37].

The 2011 questionnaire included a new variable regarding the physical fighting experienced by the students during the previous 12 months. We investigated different types of factors associated with physical fighting: demographic (gender), social (relationship with parents, parental control, emotional support and relationship with friends and their support), school performance (grades, skipped classes) and substance use (marijuana use, smoking and binge drinking).

The present study has demonstrated that the combination of male gender, binge drinking during the previous 30 days, having serious problems with friends, parent(s) who do not know where and with whom the adolescents spend their evenings, poor parental caring, low school grades, and high truancy were predictive of physical fighting in this adolescent population. However, the results do not provide information about a causal relationship.

Similar to other studies [16,39,40,41,42,43], physical fighting was more prevalent in boys than in girls. Boys usually engage in undisguised violence to gain influence, money or power. Girls resort to relational aggression and may become violent when it comes to emotional situations, such as peer and/or romantic relationships, family arguments or outsiders’ instigation [44,45,46].

According to our findings, students with poor school performance (grades between 6 and 6.99) were the most exposed to experience physical fighting compared to the highest school performance (grades between 9 and 10). The multivariate analysis showed they were three times more likely to engage in physical fight (OR = 2.16, 95%CI: 1.29–3.62, *p* = 0.002). This is similar to other studies’ findings [47,48].

Absenteeism has also been found to be associated with youth violence [14,49,50]. In our logistic regression model, students who have been missing 5–6 days from school in the last 30 days were almost seven times more likely to engage in physical fighting (OR = 6.71, 95%CI: 2.20–20.44, *p* < 0.001).

The development of bullying and victimization might be influenced by individual and family factors. Aggressive behavior research has shown that children’s socialization experiences within the family have a major role in aggressive behaviors development [36]. The following family influences on the development of aggression have been studied: family demographics (income, family type), parenting techniques (punitive and inconsistent discipline), and relationships between parent and child (positive and negative interactions) [37].

We found that the adolescents who are more likely to experience physical fights (OR = 2.22, 95%CI: 1.47–3.36, *p* < 0.001) usually have parents who rarely know with whom their children spend their evenings. Poor parental care and serious problems with friends were another two important predictors in our model.

Certainly, a wide variety of factors contribute to today’s adolescents’ exposure to violent behaviors, including family structure, social environment and peer behavior. Two of the most common correlates of violent behavior are alcohol and drug use [51,52]. For example, alcohol may suppress inhibitions against violent behavior or may affect the brain in such a way as to produce aggressive behaviors [53,54]. A competing theory proposes the reverse causal relationship, i.e., people who plan on being violent may drink to give themselves courage or an excuse for the violence [55,56,57]. Finally, a third theory states that drug use, alcohol use and violence are all outcomes of an unobserved third factor, for example, a risk-taking personality [39,51,58]. Risk taking is frequent during adolescence, and is associated with adverse outcomes including substance use. It is likely to be influenced by an individual’s cognitive development, social development, and experiences with dangerous situations [59]. The inability to recognize warning signs in dangerous situations can make drinkers easy targets for perpetrators [52]. Our study showed that alcohol consumption and drug use is a significant predictor for developing an aggressive behavior.

We observed declines in almost all aggressive behaviors in 2011 compared to 2007. This trend is also confirmed by the two HBSC studies that took place in the same periods in Romania [32,33]. After gaining access to the EU in January 2007, new legislation has been enacted and previous rules have been reinforced in areas related to youth violence. For instance, the Romanian Parliament adopted two laws on improving safety in schools: Law No. 35/2007 and Law No. 29/2010 amending and supplementing Law No. 35/2007 [60,61]. This new legislation may have played a role in reducing violence, but there is no proof of causality.

### Strengths and Limitations of the Study

There are certain limitations of this study that must be considered when interpreting the results. First, the findings reported here are only relevant to high school students from Romania who turn 16 in the calendar year of the survey and may not be generalized to other adolescents in the same age group that are not included in scholastic institutions. Second, survey methods are frequently criticized because they rely on the validity of self-reporting of sensitive and highly stigmatized behavior, thus error based on self-reported behavior might have been generated. Third, adolescents who were not available to complete the questionnaire due to truancy or dropout are likely to be at higher risk for aggressive behavior and other risk behaviors. Despite the limitations mentioned above, the study has strengths. We used a standardized questionnaire employed in other European countries in similar settings. The prevalence estimates we obtained are likely to closely represent the aggressive behavior prevalence amongst adolescents going to school, as we used probability methods for selecting the sample.

## 5. Conclusions

Physical fighting amongst the young adolescents that we evaluated was higher than the prevalence of physical fighting for the entire European ESPAD database [38], and was associated with several factors. A combination between male gender, binge drinking, problematic relationships with friends and family members, low school grades, absenteeism was found to be associated to the violent behaviors of adolescents. The development of a theoretical model which separates problem behaviors from adolescent experimental or risk-taking behaviors might be useful for future evaluations. The novelty of this study lies in analyzing patterns of associations, using a large sample with national representation. These findings may be useful to support and guide policy makers regarding the improvement and implementation of strategies to further prevent aggressive behaviors in teenagers. As in other European countries, Romania managed to reduce aggressive behaviors among high school students. New legislation may have played a role in reducing violence, but there is no proof of causality. The Ministry of Education encouraged the development of partnerships between representatives of the County School Inspectorates and the County Police Inspectorates to fight against violence in schools. In addition, the increase of alcohol excise played an important role, especially for children with limited access to their parents’ funds; this was coupled with the banning of alcohol advertising and clear rules for TV content for children and youth. Concomitantly, various guides concerning violence prevention in school were published. A school intervention strategy must provide a detailed presentation of the objectives pursued, including the expected results, the activities to be carried out, the actors involved and their responsibilities, the time horizon, the necessary resources, monitoring and evaluation modalities. These interventions should provide students and teachers information about violence, change the way adolescents feel and think about it, and teach non-violent skills in order to resolve disputes. Skill enhancement training with parents could be an important factor in controlling violence and creating a stronger family bond. Parent-skill and family-relationship approaches, providing caregivers with support and teaching communication skills, might offer problem-solving techniques and behavior-management skills. Additionally, the school psychologists should provide therapy sessions for students in order to strengthen their problem-solving skills and resistance to negative peer influence.

## Figures and Tables

**Table 1:** Factors associated with physical fight experienced by Romanian students during the last 12 months determined by univariate analysis for the 2011 survey.

Types of Factors	Variables	OR	95% CI		*p* Value
			Lower Limit	Upper Limit	
**Demographic**	**Gender**				
	Female	1 Ref			
	Male	1.95	1.67	2.29	<0.001
**Social: parents**	**Relationship with mother**				<0.001
	Very satisfied	1 Ref			
	Satisfied	1.50	1.25	1.79	<0.001
	Neither nor	1.94	1.37	2.76	<0.001
	Not so satisfied	1.31	0.91	1.87	0.142
	Not at all satisfied	1.95	1.09	3.47	0.021
	There is no such person	6.36	2.29	17.68	<0.001
	**Relationship with father**				<0.001
	Very satisfied	1 Ref			
	Satisfied	1.33	1.10	1.60	0.002
	Neither nor	1.57	1.14	2.16	0.005
	Not so satisfied	1.63	1.21	2.20	0.001
	Not at all satisfied	1.72	1.16	2.54	0.006
	There is no such person	1.69	1.07	2.68	0.022
	**Parents know where adolescents spend their Saturday nights**				<0.001
	Always	1 Ref			
	Quite often	1.99	1.64	2.42	<0.001
	Sometimes	2.84	2.22	3.36	<0.001
	Usually do not know	2.74	1.86	4.03	<0.001
	**Parents know with whom adolescents are with in the evenings**				<0.001
	Almost always	1 Ref			
	Often	2.10	1.68	2.62	<0.001
	Sometimes	2.52	1.95	3.26	<0.001
	Seldom	3.30	2.46	4.41	<0.001
	Almost never	2.01	1.54	2.63	<0.001
	**Parents know where adolescents are in the evenings**				<0.001
	Almost always	1 Ref			
	Often	1.98	1.59	2.47	<0.001
	Sometimes	2.47	1.90	3.23	<0.001
	Seldom	3.60	2.64	4.90	<0.001
	Almost never	1.92	1.43	2.58	<0.001
	**Caring from parents**				<0.001
	Almost always	1 Ref			
	Often	1.77	1.45	2.15	<0.001
	Sometimes	1.86	1.46	2.35	<0.001
	Seldom	2.66	1.90	3.74	<0.001
	Almost never	2.14	1.47	3.11	<0.001
	**Emotional support from parents**				<0.001
	Almost always	1 Ref			
	Often	1.72	1.41	2.10	<0.001
	Sometimes	1.87	1.48	2.38	<0.001
	Seldom	2.01	1.43	2.82	<0.001
	Almost never	1.86	1.27	2.72	0.001
	**Serious problems with parents**				<0.001
	No	1 Ref			
	Yes	2.02	1.71	2.39	<0.001
**Social: friends**	**Relationship with friends**				0.001
	Very satisfied	1 Ref			
	Satisfied	1.15	0.98	1.36	0.086
	Neither nor	1.70	1.20	2.40	0.002
	Not so satisfied	1.14	0.75	1.73	0.537
	Not at all satisfied	2.30	1.02	5.20	0.038
	There is no such person	3.90	0.71	21.44	0.091
	**Caring from best friend**				<0.001
	Almost always	1 Ref			
	Often	1.26	1.03	1.54	0.022
	Sometimes	1.29	1.03	1.60	0.022
	Seldom	1.62	1.22	2.16	<0.001
	Almost never	1.38	0.97	1.95	0.068
	**Emotional support from best friend**				0.001
	Almost always	1 Ref			
	Often	1.46	1.19	1.78	<0.001
	Sometimes	1.24	0.99	1.54	0.051
	Seldom	1.62	1.18	2.22	0.002
	Almost never	1.39	0.98	1.97	0.062
	**Serious problems with friends**				<0.001
	No	1 Ref			
	Yes	2.11	1.79	2.48	<0.001
**School performance**	**Grades**				<0.001
	9–10 (highest)	1 Ref			
	8–8.99	1.82	1.46	2.27	<0.001
	7–7.99	2.50	1.98	3.15	<0.001
	6–6.99	3.35	2.50	4.49	<0.001
	5–5.99	3.02	2.02	4.52	<0.001
	<5	1.09	0.29	4.09	0.892
	**Skipped classes last 30 days**				<0.001
	None	1 Ref			
	1 day	1.81	1.48	2.21	<0.001
	2 days	2.57	1.96	3.36	<0.001
	3–4 days	3.39	2.46	4.67	<0.001
	5–6 days	5.97	3.44	10.37	<0.001
	≥7 days	6.24	3.98	9.80	<0.001
**Substance use**	**Marijuana lifetime use**				<0.001
	No	1 Ref			
	Yes	2.78	2.06	3.75	<0.001
	**Current smoking**				<0.001
	No	1 Ref			
	Yes	2.46	2.07	2.92	<0.001
	**Binge drinking last 30 days**				<0.001
	No	1 Ref			
	Yes	2.91	2.46	3.45	<0.001

**Table 2:** Factors associated with physical fight experienced by Romanian students during the last 12 months determined by multivariate analysis for the 2011 survey.

Types of Factors	Variables	OR *	95% CI		*p* Value
			Lower Limit	Upper Limit	
**Demographic**	**Gender**				<0.001
	Female	1 Ref			
	Male	1.79	1.39	2.29	
**Social: parents**	**Parents know with whom adolescents are in the evenings**				<0.001
	Almost always	1 Ref			
	Often	1.64	1.19	2.26	0.002
	Sometimes	1.76	1.12	2.73	0.011
	Seldom	2.22	1.47	3.36	<0.001
	Almost never	1.42	0.88	2.31	0.146
	**Caring from parents**				<0.001
	Almost always	1 Ref			
	Often	1.26	0.95	1.68	0.113
	Sometimes	1.40	0.96	2.05	0.082
	Seldom	2.63	1.57	4.41	<0.001
	Almost never	2.17	0.88	5.39	0.085
**Social: friends**	**Serious problems with friends**				<0.001
	No	1 Ref			
	Yes	1.75	1.37	2.24	<0.001
**School performance**	**Grades**				<0.001
	9–10 (highest)	1 Ref			
	8–8.99	1.44	1.07	1.94	0.016
	7–7.99	1.68	1.19	2.38	0.002
	6–6.99	2.16	1.29	3.62	0.002
	5–5.99	0.67	0.30	1.48	0.320
	<5	3.75	0.08	179.89	0.472
	**Skipped classes last 30 days**				<0.001
	None	1 Ref			
	1 day	1.46	1.13	1.89	0.004
	2 days	1.70	1.14	2.52	0.008
	3–4 days	1.97	1.23	3.15	0.004
	5–6 days	6.71	2.20	20.44	<0.001
	≥7 days	3.28	1.72	6.27	<0.001
**Substance use**	**Binge drinking last 30 days**				<0.001
	No	1 Ref			
	Yes	2.08	1.60	2.70	<0.001

* Adjusted for all other variables in the model.

**Table 3:** Violent behavior: 2007 compared to 2011 among Romanian students.

Year	2007		2011		Significance	
Characteristic	Not at all, *n* (%)	Once or more Times, *n* (%)	Not at all, *n* (%)	Once or more Times, *n* (%)	Chi Square	*p* Value
Hit teacher	2232 (98.20)	42 (1.80)	2685 (98.38)	52 (1.62)	0.018	0.891
Males	963 (96.70)	36 (3.30)	1223 (97.53)	35 (2.47)	1.233	0.267
Females	1269 (99.54)	6 (0.46)	1462 (99.06)	17 (0.94)	3.810	0.051
Got mixed into a fight where a group of friends were against another group	1853 (81.63)	408 (18.37)	2273 (83.49)	454 (16.51)	1.687	0.194
Males	740 (74.25)	251 (25.75)	955 (76.71)	297 (23.29)	0.772	0.379
Females	1113 (88.28)	157 (11.72)	1318 (89.0)	157 (11.0)	1.988	0.158
Participated into a group fight	1763 (77.16)	503 (22.84)	2239 (82.86)	470 (17.14)	18.434	<0.001
Males	707 (70.03)	285 (29.97)	943 (76.0)	298 (24.0)	6.358	0.012
Females	1056 (83.53)	218 (16.47)	1296 (88.39)	172 (11.61)	16.271	<0.001
Hurt someone badly	2070 (90.57)	196 (9.43)	2504 (92.23)	221 (7.77)	0.470	0.493
Males	862 (85.73)	132 (14.27)	1093 (87.69)	158 (12.31)	0.207	0.648
Females	1208 (94.93)	64 (5.07)	1411 (95.91)	63 (4.09)	0.887	0.346
Weapon use	2223 (98.13)	42 (1.87)	2659 (97.79)	70 (2.21)	2.851	0.091
Males	960 (96.48)	35 (3.52)	1204 (96.46)	50 (3.54)	0.336	0.562
Females	1263 (99.62)	7 (0.38)	1455 (98.87)	20 (1.13)	4.537	0.033
Group teasing	1119 (47.16)	1152 (52.84)	1511 (54.07)	1230 (45.93)	17.057	<0.001
Males	480 (44.09)	521 (55.91)	689 (53.74)	572 (46.26)	9.992	0.002
Females	639 (49.93)	631 (50.07)	822 (54.35)	658 (45.65)	7.494	0.006
Group bruising	1728 (75.55)	537 (24.45)	2134 (77.98)	601 (22.02)	2.119	0.145
Males	731 (72.09)	265 (27.91)	938 (74.4)	321 (25.61)	0.356	0.551
Females	997 (78.68)	272 (21.32)	1196 (80.90)	280 (19.1)	2.578	0.108
Gang attacking	1764 (78.03)	499 (21.97)	2230 (81.72)	503 (18.28)	10.262	0.001
Males	742 (74.01)	252 (25.99)	963 (76.55)	293 (23.45)	1.238	0.266
Females	1022 (81.66)	247 (18.34)	1267 (85.92)	210 (14.08)	13.541	<0.001
Individual attacking	1620 (70.80)	629 (29.20)	2178 (80.31)	546 (19.69)	42.865	<0.001
Males	614 (60.22)	374 (39.78)	917 (73.45)	335 (26.55)	31.434	<0.001
Females	1006 (80.32)	255 (19.68)	1261 (85.88)	211 (14.12)	16.646	<0.001
Group teased	1248 (53.15)	1016 (46.85)	1732 (63.04)	1004 (36.96)	34.430	<0.001
Males	515 (48.84)	481 (51.16)	783 (62.80)	478 (37.20)	24.568	<0.001
Females	733 (57.03)	535 (42.97)	949 (63.23)	526 (36.77)	12.263	<0.001
Group bruised	1953 (85.56)	302 (14.44)	2431 (89.50)	296 (10.50)	7.527	0.006
Males	787 (78.02)	204 (21.98)	1065 (85.69)	192 (14.31)	10.769	0.001
Females	1166 (92.38)	98 (7.62)	1366 (92.62)	104 (7.38)	0.457	0.499
Gang attacked	1856 (82.63)	388 (17.37)	2358 (87.08)	365 (12.92)	14.445	<0.001
Males	757 (75.85)	230 (24.15)	1035 (83.24)	215 (16.76)	12.890	<0.001
Females	1099 (88.76)	158 (11.24)	1323 (90.20)	150 (9.80)	3.858	0.049
Individual attacked	1571 (68.07)	683 (31.93)	2122 (78.91)	588 (21.09)	47.825	<0.001
Males	574 (56.10)	416 (43.90)	876 (71.22)	371 (28.78)	36.427	<0.001
Females	997 (78.83)	267 (21.17)	1246 (85.18)	217 (14.82)	18.383	<0.001
